# COVID-19: Marking the Gaps in Migrant and Refugee Health in Some Massive Migration Areas

**DOI:** 10.3390/ijerph182312639

**Published:** 2021-11-30

**Authors:** Stephen A. Matlin, Ozge Karadag, Claudio R. Brando, Pedro Góis, Selma Karabey, Md. Mobarak Hossain Khan, Shadi Saleh, Amirhossein Takian, Luciano Saso

**Affiliations:** 1 Institute of Global Health Innovation, Imperial College London, South Kensington, London SW7 2AZ, UK; s.matlin@imperial.ac.uk; 2 Global Health Centre, Graduate Institute of International and Development Studies, 1202 Geneva, Switzerland; 3 Center for Sustainable Development, Earth Institute, Columbia University, New York, NY 10115, USA; 4 Education and International Relations Office, Hospital Universitario San Ignacio, Bogotá 11001, Colombia; crbrando@husi.org.co; 5 Faculty of Economics, University of Coimbra, 3004-512 Coimbra, Portugal; pedro.gois@uc.pt; 6 Istanbul Faculty of Medicine, Istanbul University, Fatih, Istanbul 34093, Turkey; selmakarabey@gmail.com; 7 Department of Social Relations, Faculty of Liberal Arts and Social Sciences, East West University, Dhaka 1212, Bangladesh; mmhkhan@ewubd.edu; 8 Global Health Institute, American University of Beirut, Beirut 1107 2020, Lebanon; ss117@aub.edu.lb; 9 Health Equity Research Center (HERC) and Department of Global Health & Public Policy, School of Public Health, Tehran University of Medical Sciences, Tehran 1417613151, Iran; takian@tums.ac.ir; 10 Department of Physiology and Pharmacology Sapienza University of Rome, 00185 Rome, Italy; luciano.saso@uniroma1.it

**Keywords:** COVID-19, migrant and refugee health, massive migration, health framework, structural factors, health determinants, human security, health gaps, sustainable development goals

## Abstract

The health of migrants and refugees, which has long been a cause for concern, has come under greatly increased pressure in the last decade. Against a background where the world has witnessed the largest numbers of migrants in history, the advent of the COVID-19 pandemic has stretched the capacities of countries and of aid, health and relief organizations, from global to local levels, to meet the human rights and pressing needs of migrants and refugees for access to health care and to public health measures needed to protect them from the pandemic. The overview in this article of the situation in examples of middle-income countries that have hosted mass migration in recent years has drawn on information from summaries presented in an M8 Alliance Expert Meeting, from peer-reviewed literature and from reports from international agencies concerned with the status and health of migrants and refugees. The multi-factor approach developed here draws on perspectives from structural factors (including rights, governance, policies and practices), health determinants (including economic, environmental, social and political, as well as migration itself as a determinant) and the human security framework (defined as “freedom from want and fear and freedom to live in dignity” and incorporating the interactive dimensions of health, food, environmental, economic, personal, community and political security). These integrate as a multi-component ‘ecological perspective’ to examine the legal status, health rights and access to health care and other services of migrants and refugees, to mark gap areas and to consider the implications for improving health security both for them and for the communities in countries in which they reside or through which they transit.

## 1. Introduction

The exceptional pressures created by the COVID-19 pandemic have revealed weaknesses at all levels in health and pandemic preparedness systems. They have not only exposed but in many cases exacerbated inequalities and gaps in health rights, access and treatment experienced by a number of disadvantaged groups. During the pandemic, the situation of many migrants and refugees, whose access to health services is often greatly restricted in any case [1], has exemplified such inequities. It has also provided a signal of the weaknesses and gaps in capacities of public health systems to prepare for and respond to infectious disease challenges with measures that provide adequate protection equitably for all [2,3].

A World Health Summit M8 Alliance Expert Meeting on the impact of COVID-19 on migrant and refugee health [4] was organized in collaboration with the Association of Academic Health Centers International on 22 February 2021. It reviewed situation summaries related to diverse migration contexts in some regions and countries that have hosted very high levels of migration in recent years, including large numbers (1 m or more) of refugees. These summaries highlighted considerable disparities and gaps, within and between countries and regions, that reflected the operation of a range of drivers, determinants and impediments. They provide a spotlight to examine the legal status, health rights and access to health care and other services of migrants and refugees. They help to mark gap areas and consider the implications for improving health security for migrants and refugees and also for the communities in countries in which they reside or through which they transit.

The present article draws on evidence from the M8 Alliance Expert Meeting and other sources. It considers how a number of structural factors—including governance, policy and practice—interact with a range of health determinants and components of human security, as well as the impacts of COVID-19, to affect the health of migrants and refugees in areas of massive migration. This multi-factor approach marks many inequities and gaps that need to be addressed.

## 2. Methods

**Selection of country examples:** The number of people living outside their country of origin rose to 281 million in 2020, representing about 3.6 per cent of the world’s population. Refugees accounted for 12 per cent of all international migrants in 2020, up from 9.5 per cent in 2000, an increase involving a doubling in numbers from 17 to 34 million. The distributions of the two groups are extremely different, with nearly two thirds of all international migrants living in high-income countries, while 31 per cent live in middle-income countries and around four percent in low-income countries. However, low- and middle-income countries hosted 80 per cent of the world’s refugees in 2020, who comprised around three per cent of all international migrants in high-income countries, compared to 25 per cent in middle-income countries and 50 per cent in low-income countries [5].

The M8 Alliance Expert Meeting selected examples of middle-income countries (Colombia, Iran, Jordan, Lebanon and Turkey) which, in recent years, have been hosts to large numbers of international migrants, including more than 1 million refugees, to examine their particular situations and how these have been impacted by the COVID-19 pandemic.

An additional middle-income country, Bangladesh, was included to exemplify a particular feature of the impact of the COVID-19 pandemic. While becoming host to nearly 900,000 Rohingya refugees from Myanmar in the last few years, Bangladesh has for long been the source of international migrant labor, including to Middle East and Persian Gulf states. During the COVID-19 pandemic, many of these migrants were forced to return home, creating new economic and social problems for the country.

**Information sources:** This article gathered information from three main sources: (1) the expert summaries of specific country and regional situations presented by the discussants in the M8 Alliance Expert Meeting and which are available in the recording of the event [4]; (2) peer-reviewed literature identified by searches for reports on migrant and refugee status and health related to the countries and regions of interest, and (3) reports from international agencies concerned with the status and health of migrants and refugees.

## 3. Framing the Analysis

### 3.1. People and Promises

When people move across an international border to live outside their own country, their access to rights and services in general and to health-related aspects in particular, is heavily influenced by their status as ‘documented’ or ‘undocumented’ and whether they are recognized as being ‘migrants’, ‘refugees’ or ‘asylum seekers’, since commitments by states are usually based on these categories [6,7,8,9,10,11]. The 21st Century has seen some of the largest migrations in history [12] stemming, among other causes, from economic, environmental political, socio-demographic factors and conflicts and disasters [13,14,15]. In particular, the period 2014–2015 witnessed the largest and most rapid escalation ever in the number of people being forced from their homes, creating the highest level of displacement since World War II. The number of international migrants reached 281 million people in 2020, representing about 3.6 per cent of the world’s population and up from 173 million in 2000 and 221 million in 2010 [5,16]. The United Nations High Commissioner for Refugees (UNHCR) reported [17] that at the end of 2019, as well as 45.7 million internally displaced people, there were 26 million refugees and 4,2 million asylum seekers. The total of displaced people amounted to about 1% of the world’s population. Three quarters of people displaced internationally are hosted in neighboring (predominantly low- and middle-income) countries.

### 3.2. Health: Rights, Provisions and Practices

The New York Declaration adopted by the UN General Assembly in 2016 [8] deals with a number of aspects of health. It encourages states to address the specific health care needs experienced by migrant and mobile populations, as well as by refugees and crisis-affected populations. Areas covered include HIV prevention and treatment, combatting sexual and gender-based violence, access to sexual and reproductive health care services, and improving integration and inclusion in access to health care.

The 2018 UN Global Compact for Safe, Orderly and Regular Migration [9] commits countries to collect and utilize accurate and disaggregated data as a basis for evidence-based policies, including in health. Countries also commit to provide migrant workers engaged in remunerated and contractual labor with the same labor rights and protections extended to all workers in the respective sector, including the right to the highest attainable standard of physical and mental health. The Compact requires countries to develop gender-responsive migration policies to address, among others, health care and psychological and other counselling services; to protect unaccompanied and separated children at all stages of migration, including providing access to health-care services, including mental health; and to reduce negative and potentially lasting effects of detention on irregular migrants, including by granting access to basic health care. Furthermore, there are commitments to ensure access to health care for all children, including those with their families and those unaccompanied or separated; to incorporate the health needs of migrants into national and local health-care policies and plans, with training of health-care providers on culturally sensitive service delivery; and to develop national short-, medium- and long-term policy goals regarding the inclusion of migrants in, among others, health.

The 2018 UN Global Compact on Refugees [10] deals only briefly with health. It affirms that, at the request of concerned states, UNHCR and relevant stakeholders will contribute resources and expertise to support timely health assessments of new arrivals. States and relevant stakeholders will contribute resources and expertise to expand and enhance the quality of national health systems to facilitate access by refugees and host communities, including women and girls; children, adolescents and youth; older persons; those with chronic illnesses, including tuberculosis HIV, and mental health issues; survivors of trafficking in persons, torture, trauma or violence, including sexual and gender-based violence; and persons with disabilities. Disease prevention, immunization services, and health promotion activities are encouraged, as are pledges to facilitate affordable and equitable access to adequate quantities of medicines, medical supplies, vaccines, diagnostics, and preventive commodities.

In 2019, the World Health Assembly adopted a five-year Global Action Plan to Promote the Health of Refugees and Migrants presented by the World Health Organization (WHO) [18]. This asserts values (human rights, non-discrimination, gender sensitivity), goals (quality health care, occupational safety, and public health) and key tools (information systems, communication, and advocacy for migrant rights). It aims to achieve universal health coverage and the highest attainable standard of health for refugees and migrants, as well as for host populations, through full and equal inclusion of migrants in health systems [19].

The instruments outlined above ascribe important roles to international cooperation and the complementary functions of global and regional bodies. However, they emphasize that primary responsibility rests with States to respond to the health needs of migrants and refugees arriving in their own countries and to support those trying to meet the health needs of migrants and refugees in camps or transit locations on the way to their destinations. The diverse country responses therefore need to be examined in identifying examples of good practice to disseminate, as well as gaps in migrant and refugee health. This examination includes three dimensions: (1) national policies put in place to address the health of migrants and refugees; (2) systems and resources (including financial and human, with education and training as an important element of the latter) instituted to implement the policies; and (3) consultation, monitoring and evaluation mechanisms (inclusive of the migrants and refugees themselves) instigated to ensure that the policies are being delivered and are meeting the health needs of the migrants and refugees.

### 3.3. COVID-19 as an Additional Stress

During the pandemic, existing social inequalities were deepened and new areas of inequality created [3]. High levels of inequality have increased vulnerability to COVID-19 and lowered social cohesion, generating less social trust and more political polarization. As data have accumulated, it has become apparent that the pandemic affects different population groups in an unequal way [20]. Some groups have been deeply affected by the pandemic, such as women (increased gender-based inequalities, violence against women, workload at home and unwanted pregnancies, as well as withdrawal from working life), children (increased inequalities in access to education, increased child labor, neglect and abuse), the elderly (increased isolation, difficulty in accessing services) and migrants (changing migration policies, admission conditions, deportations, increased stigma and discrimination). Karadag and Karabey point out that COVID-19 has evolved into a worldwide social disease which has a strong relationship with social factors [20].

The COVID-19 pandemic has impacted the practical and academic approaches to migration and health. Recent studies especially focus on increasing vulnerabilities of migrants, deepening inequalities and stigma, as well as the importance of intersectionality and why all policies and practices need to include migrant populations in order to leave no one behind [2,21,22,23]. This article attempts to address the current debate to build forward better in terms of strengthening migrant health policies and practices and having more inclusive pandemic preparedness plans for the future.

### 3.4. Perspectives of Enquiry

**Figure 1:**
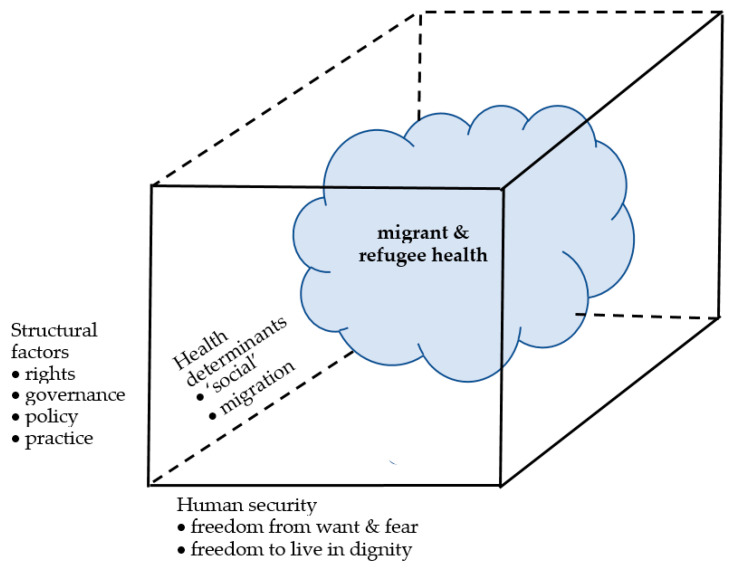
Perspectives contributing to migrant and refugee health in the COVID-19 pandemic.

Several frameworks have been suggested for promoting the health of migrants and refugees [1,24,25,26,27,28,29,30,31]. However, these are generally selective in approach and rooted in sectors, stages of migration, health issues, orientation towards policy, practice or outcome and none takes a comprehensive view. In this exploration to identify gaps, we have therefore adopted a multi-factor approach (an ‘ecological perspective’). This combines three broad, complementary and overlapping perspectives considered to be of particular significance (Figure 1), from which to view the health situation of migrants and refugees in massive migration areas during the COVID-19 pandemic:

- Structural factors: Components include rights, governance, policies and practices.
- Health determinants: Frameworks based on the ‘social’ (which includes economic, environmental, social and political) determinants of health have been applied to the health of migrants and refugees. Moreover, migration itself has been recognised as a determinant, with risks to the health of migrants that may arise at every stage along their journeys, from before the migration process starts, during travel and at transit and destination point [1,32,33,34,35].
- Human security: The 1994 Human Development Report (HDR) of the United Nations Development Programme (UNDP) [36] replaced the traditional interpretation of security as relating to national/territorial security with a new one centred on the security of people. The new concept of human security, defined [37] as “*freedom from want and fear and freedom to live in dignity”* simultaneously broadened the focus of security itself, *while also emphasising the role of actors beyond the nation state and the vital importance of collective international responsibility and action, recognizing* the multi-sectoral interconnectivity of factors operating across the spectrum of social, economic, and cultural affairs [38,39,40]. The 1994 HDR listed seven main, interactive and interconnected categories of threats to human security: health, food, environmental, economic, personal, community and political.

## 4. Results and Discussion

### 4.1. Some Regional and Country Experiences of Massive Migration

The **Arab region** hosts over 38 million migrants and refugees and around 15 million internally displaced persons, while over 29 million people from Arab countries are living outside of their countries of origin [41]. By 2021 the conflict in Syria alone had displaced 6.7 million people internally and more than 6.6 million externally [42], of whom 5.6 million were living in nearby countries within (e.g., Jordan, Lebanon) or just beyond (e.g., Turkey) the region itself. Many of the refugee source and host countries are fragile, including in their health care systems [43]. There are global implications in terms of direct (e.g., migration to the North) and indirect (e.g., costs to the UN system and global community) burdens. This emphasizes the need for global and regional approaches and solutions, rather than relying solely on country-specific ones that may be politically challenging and are sometimes relatively costly and/or less effective.

The massive migrations within and beyond the Arab region illustrate challenges in both general and health-specific governance gaps. In the absence of a unified, overall governance mechanism for responding at global and regional levels to migrant and refugee situations, Inter-State Consultation Mechanisms on Migration (ISCM) began to be established in the mid-1980s. By 2018 there were 30 ISCMs covering every region of the world and with a total membership of 160 states [44,45]. The regional consultative processes on migration (RCPs) may address a wide range of issues, including migration and health [46]. As well as IOM, these mechanisms are supported by global (e.g., UNHCR) and regional (e.g., League of Arab States) partner agencies. The Arab Regional Consultative Process on Migration and Refugees Affairs [47] was established as one such discussion and cooperation platform in 2015 and is permanently chaired by the League of Arab States. Interregional forums on migration [48] (IRFs) bring together member states from two or more regions and have been used, for example, to discuss migration corridors. However, the sometimes-conflicting policy interests of member States in an IRF, as well as substantive differences in migration dynamics, interests and desired outcomes, may make it more difficult to reach consensus. There does not appear to have been an IRF focusing on the Arab and European regions. However, the ‘5 + 5 Dialogue on Migration in the Western Mediterranean’ IRF involves North African countries that are members of the Arab region and EU countries and its current thematic focus includes migration and health [49]. The Budapest Process [50] bridges Europe and Asia and has a current focus on irregular migration and labour migration—but, while having Turkey as a member and permanent chair, includes no members of the Arab Regional Consultative Process.

At the regional level, efforts to coordinate health provisions are led by the Eastern Mediterranean Regional Office (EMRO) of WHO (whose membership of 22 countries largely overlaps with that of the League of Arab States, while also including Afghanistan, Iran and Pakistan). EMRO is the WHO region with the largest presence of refugees and displaced populations. In 2019, the region hosted 66% (16.7 million) of the total number of refugees worldwide and 33% (1 million) of the world’s asylum seekers. In addition, there was a Palestinian refugee population of 5.2 million people living in camps and host communities between Jordan, Lebanon, Syrian Arab Republic and in the West Bank and Gaza Strip. An Eastern Mediterranean Region action plan [51] to promote the health of migrants, refugees and displaced populations were initiated in 2019 has yet to be finalized and was overtaken by discussion of the COVID-19 pandemic [52,53,54].

At the country level, major differences in governance, policy and practice affect the health of migrants and refugees in and from the Eastern Mediterranean region and neighboring countries [55]. In Jordan, health service coverage of the general population, provided by a mix of public and private sector approaches, is far from complete and largely unaffordable by the poorest sections of the population, of whom more than one-quarter are not insured [56]. For refugees, the extent of coverage is status- and circumstance-dependent [57]. With an overall population of about 10 million, the country has more than two million Palestinian refugees, most having full citizenship while ten refugee camps serviced by the United Nations Relief and Works Agency for Palestine Refugees in the Near East (UNRWA) accommodate nearly 370,000 Palestine refugees. Part of UNRWA’s mandate is to provide basic health services for registered Palestinian refugees in Jordan and other countries [58,59,60], both inside and outside its camps, including Palestinians originally displaced to Syria and then more recently from there due to the Syrian conflict. A systematic review found positive impacts of the UNRWA health services on the health of Palestinian refugees in Jordan, compared with the non-refugee population [61] but gaps in access, uptake and/or provision have been identified [62]. Examples included systemic problems within UNRWA’s health centers, cost as a barrier to accessing hospital services and weaknesses in mental health services (both resource and social determinant barriers) [63] and ante-natal services (health system, personal and social determinants) [64].

In the study of the health status of refugees from Syria in Jordan, it was noted that the Palestinian refugees compared themselves unfavorably with others who were covered by UNHCR [62]. An EMRO report [65] observed that the large influxes of Syrian refugees into Jordan had overshadowed other refugee populations (e.g., from Iraq, Somalia, Sudan and Yemen), with donor responses focusing on the Syrian humanitarian crisis. As well as fragmented responsibility, this illustrates aspects of the sectoral challenge [66] with dimensions including the difficulties arising from the fact that many health issues originate in sectors outside health. Challenges arise (for both donors and specialized agencies), in the integration of humanitarian (sometimes short-term) and development (usually long-term) responses [67] which are often modulated by political positions. As well as Palestinian refugees, there are about 0.75 million other refugees in Jordan, among them being about 660,000 Syrian refugees, with over 80 percent of these living in urban centers while the remaining 20 percent of Syrian refugees live in two refugee camps, established by the Jordanian authorities and managed by UNHCR. This agency co-chairs the Health Working Group in Jordan with WHO, providing information, advice and advocacy to high-level decision-making bodies in Jordan. UNHCR and partners have adopted and advocate for the “One Refugee” approach for all persons of concern, Syrian and non-Syrian, in all sectors and services in Jordan. The approach seeks to reduce and ultimately eliminate the acknowledged differences in rights and services based on nationality [68].

Prior to the Syrian conflict, **Lebanon** had a healthcare system dominated by the private sector and angled towards hospital-based curative care rather than primary and preventive health measures, with half the population lacking formal health insurance [69]. In 2013, the World Bank [70] described the Lebanese health and social security systems as “weak, fragmented and poorly targeted” and health inequalities continue to be strongly influenced by local socio-political factors and factionalism [71,72]. Lebanon has hosted Palestinian refugees since 1948 and these people and their descendants, current numbering about 300,000, live in 12 official camps, informal gatherings and communities with Lebanese citizens, with UNRWA being the lead agency for their needs. In addition, since 2011 Lebanon has received more than 1 million refugees from Syria, making it the country with the highest number of refugees per capita of the population in the world, Lebanon also hosts about 20,000 refugees of other nationalities, including Ethiopia, Iraq and Sudan. Responsibility for health care for Syrian refugees is shared by the Ministry of Public Health, UNHCR, NGOs and humanitarian agencies. It has been government policy not to create formal refugee camps in response to the influx of the Syrian refugees, but rather to absorb them in urban locations. However, tented settlements have been spontaneously set-up throughout the country. In practice, the accommodation of most of the refugees from Syria is lacking in basic facilities and overcrowded [73]. Amidst complex, multi-sectarian social and political rivalries, security threats and economic shocks, the large-scale arrival of refugees from Syria has exposed the fragile nature of the pre-existing public health system [74,75]. It has led to increased tensions between host communities and the refugee population, who compete for the limited health resources [76]. In this complex arena, UNHCR’s role [77,78] is to facilitate and advocate for access to its persons of concern through existing services and health service providers and to monitor access to health care services.

Turkey hosts around 3.7 million refugees from Syria and, as of April 2020, there were also around 455,000 irregular migrants mainly from Afghanistan, Pakistan and Syria. According to UNHCR, there were an additional 370,000 asylum seekers and refugees under international protection in Turkey, most of whom were from Afghanistan and Iraq. Turkey is a member of WHO’s European Region (EURO) and benefits from inclusion in the Regional Office’s work, including monitoring of health status of refugees and migrants [79]. While not being within the European Union (EU), Turkey has received some support for refugees from UNHCR [80], IOM [81], ICRC [82] and IFRC [83] and also from the European Commission in collaboration with partner agencies [84], but the level of the EU’s contributions has been a point of contention [85].

One aspect of Turkey’s inclusive approach has been to train about 2000 Syrian health workers to work in a network of 178 refugee health centers throughout Turkey. The Ministry of Health has already hired over half of them to provide health services to Syrian refugees and help overcome cultural and linguistic barriers.

**The Islamic Republic of Iran** hosts another large and very protracted urban refugee situation. Conflicts in neighboring Afghanistan, extending over four decades, resulted in nearly 5 million Afghans being displaced from their country and of these, 90% are hosted by Pakistan and Iran. The latter has approximately 3 million Afghans living in the country, of whom 2.25 million are undocumented and 0.8 million are refugee card holders [86,87]. Almost all refugees in Iran live in urban settings alongside the host community, while 4% live in 20 settlements managed by the Ministry of Interior. Iran operates a Universal Public Health Insurance (UPHI) system aiming at universal health coverage (UHC) and since 2015 the government’s inclusive policies provide both refugees and other immigrants with access to health, as well as education and livelihoods opportunities [88]. However, the compulsory health insurance coverage law remains to be fully implemented, and a substantial gap between private and public medical tariffs leads to high out-of-pocket health expenditure [89]. This is gradually being addressed by the free Universal Health Coverage Fund which aims to reach UHC by 2025, although the sustainability of the UHC approach is seen as challenging [90,91]. Meanwhile there are large gaps in health status between the better-off population and the more vulnerable, including refugees and migrants [92]. UNHCR covered the costs of insurance premiums for about 100,000 vulnerable refugees enrolled in Iran’s UPHI in 2020 and is seeking to expand this further in the face of the added challenge of the COVID-19 pandemic [93]. The undocumented Afghan migrants in Iran have much more limited access to health care than registered refugees, as they are not covered by the public health insurance and face much higher charges, as well as sometimes experiencing discrimination when seeking treatment [94,95,96,97].

The Latin American region, having itself been a major destination [98] for migrants in earlier times, in recent years has become a major source of migrants and refugees. About 37 million Latin Americans (one in seven global migrants) lived outside of their native countries in 2017, including in USA, Europe and other countries in the Latin American region [99] with major drivers including economic, political and security factors. The Inter-American Commission on Human Rights (IACHR), an organ of the Organization of American States (OAS), promotes the observance and protection of human rights in the Americas and works with the Inter-American Court of Human Rights. The IACHR has instituted eight thematic rapporteurships, one of which deals with the rights of migrants, health is treated as a cross-cutting rights issue [100]. The Latin American region forms part of the membership of the Pan-American Health Organization (PAHO), which serves as the Regional Office of WHO. In its advisory role [101] it recommends strategic actions and urges “*Member States to generate health policies and programs to address health inequities that affect migrants and develop targeted interventions to reduce migrants’ health risks; improve regulatory and legal frameworks to address the specific health needs of migrants; ensure access to the same level of financial protection and health services that other people living in the same territory enjoy, regardless of their migratory status; and generate proposals at all levels for the coordination of programs and policies on health issues considered to be of common interest in border areas*.” PAHO aims to develop a global action plan on the health of refugees and migrants in the region [102].

Deteriorating conditions in several countries in this region resulted in a marked acceleration of migration in the last few years, both intra-regionally and beyond [103]. Colombia has seen large movements of people within and in both directions across its borders in recent decades. Conflicts, including those related to politics drugs and other crimes, have led to more than 4 million Colombians (about 10% of the country’s population) living abroad, including about a third of a million refugees, as well as more than 7.5 million internally displaced persons. Colombia is also on a pathway for migration for people from other countries to the south and from other regions of the world heading towards North America. The negative economic situation, lack of access to basic social needs such as healthcare and food, lack of money in cash, and political polarization have been the main drivers of emigration of over 5 million people from Venezuela [104]. Prior to the COVID-19 pandemic, countries in the region largely maintained an ‘open-door’ approach toward Venezuelans, allowing many to enter and remain with legal status on an interim basis, as well taking requests for asylum. More than 4 million Venezuelans moved to other countries in Latin America and the Caribbean, including about 1.8 million who relocated to Colombia [105], alongside the return of Colombians who had earlier migrated to Venezuela in the period when their own country was experiencing considerable violence.

The large-scale movement of Venezuelans has presented a growing health challenge in Colombia and other countries in the region [106]. A survey of migrants from Venezuela in the Latin America and the Caribbean region revealed extremely diverse experiences, including in health provisions [107]. The exodus of skilled Venezuelans, including health workers, has exacerbated the steep decline in the health system in Venezuela, while for Colombia the impacts of sudden large-scale arrivals have also included severe stressing of its health system. In February 2021, the Colombian government announced it was introducing a *ten-year* Temporary Statute of Protection to regularize the status of nearly 1 million Venezuelan migrants and provide access to public systems and services [108].

### 4.2. Impacts of the COVID-19 Pandemic

Against the background of large-scale movements of migrants and refugees that have been experienced by a number of regions of the world in the last decade, the advent of COVID-19 in late 2019 and its rapid development into a pandemic in early 2020, has generally considerably worsened both the general circumstances and specific health challenges faced by these people [109].

As with other aspects of the health of migrants and refugees, their experiences and the impacts on them of the COVID-19 pandemic have been very diverse, multi-dimensional and reflecting specific local, regional and global contexts [110,111,112]. However, commonalities across regions have included a paucity and limited reliability of data on COVID-19 infections [113] and limited availability of data that might reveal cross-correlations with factors such as education and income status, ethnicity and immigrant status; increased risks and vulnerabilities of irregular migrants, asylum seekers and refugees due to their working and accommodation conditions while living in poverty, being in crowded houses and having difficulties with social distancing; and experiencing stress-provoking factors including high levels of uncertainty, and increased stigma and discrimination against refugees and asylum seekers within host communities [114].

Migrant populations, especially refugees, asylum seekers, displaced populations and irregular migrants face specific risks from COVID-19, including difficulties in accessing reliable, linguistically and culturally appropriate information about COVID-19, as well as difficulties in accessing healthcare, education, and other services including services provided via internet and digital technologies. Substandard accommodation and hygiene conditions, economic hardships, job loss or working in informal sector under risky conditions, fear of deportation, closed borders, interruptions in asylum interviews increase these populations’ vulnerability, in addition to increased biological risks due to high prevalence of nutritional deficiencies and unmonitored chronic diseases [2,114]. These factors all require regular surveillance, research, analysis, and documentation for more evidence and needs-based policies.

As an example of an individual country response, immediately after the start of the pandemic the Turkish Government announced that COVID-19 related health services, including free access to personal protective equipment, diagnostic testing and medical treatment, would be provided free regardless of registration status, facilitating access to health services during the outbreak for irregular migrants. However, language barriers led to difficulties in accessing health information and unregistered migrants were reluctant to visit health institutions because of fears of deportation or loss of accommodation or employment [114].

A striking example of the human security concept in operation is provided by the COVID-19 pandemic. It has demonstrated how a threat to one of the seven core categories (health) of human security has led to major impacts across all of them, requiring multi-sectoral actions at local, national and global levels [115]. As well as the direct health effects that have included hundreds of millions of infections and millions of deaths [116], there have been impacts on economies amounting to trillions of dollars [117,118]. Dramatic consequences for the lives of individuals have including unemployment, impoverishment, social isolation, interruptions to diagnosis and treatment for other serious health conditions and deterioration in mental health and in access to education [119,120]. The pandemic has exposed the lack of adequate preparedness of global health security systems and often-weak capacity, readiness and robustness of national health systems to cope with a serious disease outbreak [121], with implications for achieving the UN Sustainable Development Goals (SDGs) [122,123]. Against this background, migrants and refugees have been among the most vulnerable groups, subjected to travel restrictions, lockdowns, increased stigma and discrimination, social isolation requirements that their circumstances made it difficult or impossible to meet, and limited availability of personal protection equipment and treatment opportunities and of access to the vaccines once these began to be rolled out.

Bangladesh illustrates many of the central features of the human security concept, demonstrating the interdependence of health security with other elements. In this case, particularly economic and food security are the most immediately connected, but with significant impacts also in broader personal, community and political domains. While currently hosting over 850,000 Rohingya refugees from Myanmar since the crisis began there in 2017 [124], Bangladesh has long been the source of large numbers of migrants (usually more than 750,000 per year [125]) who go abroad, mainly to the Middle East and Persian Gulf states, for work and whose remittances home are of major importance to both their families and the national economy [126,127]. The advent of COVID-19 has had major impacts on these migrants (many of whom lost their jobs and, consequently, their accommodation and right to remain in the host country) and their families [125,128,129]. At least 200,000 Bangladeshi workers are believed to have returned from overseas in February-April 2020, with more than half being in debt; while 40% of those migrant workers abroad were jobless [125,130,131]. It was estimated that remittances from overseas would decline by about 22% from 2019 to 2020 [131,132].

Many lessons can be learned from COVID-19, including that the pandemic could have been prevented if earlier warnings from scientists had been heeded. The costs of prevention would have been hundreds of times less than the costs now being borne in responding to the pandemic [117,118]. Health security both locally and globally depends on everyone being entitled and enabled to participate in national programs for prevention, diagnosis, treatment and vaccination—including migrants and refugees.

### 4.3. Marking the Gaps

The present overview of situations of migrants and refugees in areas of massive migration before and during the COVID-19 pandemic reveals gaps in health that relate to many of the elements that are included in the multi-factor framework used in this article. Examples include:

Structural factors: The present study highlights examples of deficits in all aspects of the structural factors important to ensure the health of migrants and refugees. Intrinsic gaps are seen in the extent of health coverage entitlement afforded to different individuals according to their categorization. These are further enlarged by the failure of states to incorporate globally agreed rights into national policies and to operationalize these entitlements into accessible, affordable provisions.

Noting that “*few human rights obligations are more widely disregarded than migrant health*” and that “*migrants should have equal access to national health systems, but they don’t*”, Gostin [19] has been among those who have questioned the capacity to deliver the WHO Global Action Plan to Promote the Health of Refugees and Migrants adopted by the WHA. Reasons include the spread of responsibilities among many actors, the limitations of resources provided and the necessity to operate within country contexts and financial situations and in line with national priorities and legal frameworks. Frenk and Moon [66] highlighted three persistent governance challenges in global health. These relate to (1) sovereignty (the “*inherent tension between the reality of national sovereignty and the imperative of international collective action to properly manage interdependence*” since “*the determinants of health and the means to fulfil that responsibility lie increasingly beyond the control of any one nation state*”, but “*there is no government at the global level*”); (2) sectoral issues (including how health is influenced by governance of non-health sectors); and (3) accountability (including the conflicting interests of those to whom organizations and political groupings are accountable). Onarheim and Rached [133] have drawn attention to the importance of these in relation to the health of migrants and refugees and their examination concluded that the WHO Global Action Plan relies on weak accountability mechanisms.

The situations experienced by migrants and refugees that are noted in the present article reinforce these conclusions and extend them further. They highlight examples, not just in the Global Action Plan but across all levels from global to national and local, of failures to support migrant and refugee health in which sovereignty is unclear, governance highly fragmented, sectoral responsibilities (e.g., between health and humanitarian support) divided and accountability incomplete. Compliance with international instruments to which a state has acceded—especially when non-binding or effectively non-enforceable—largely depends on political willingness in dynamically changing circumstances and may be heavily modulated by domestic political agendas as well as by international positioning. As Gilbert [134] observed regarding the Global Compact on Refugees, it “*is not legally binding, but it gives rise to commitments by the international community as a whole*”. This inherent tension is seen to unfold in diverse ways in the regional and country responses to health in the context of massive migration.

**Health determinants:** Given the ways that health conditions and vulnerabilities of migrants and refugees change with circumstances, a dynamic analysis is important. This needs to consider temporal events and cumulative impacts of different determinants at different stages and phases [1,34]. For example, a systematic review [135] to identify the structural and social determinants which put migrants and refugees at risk of poor sexual and reproductive health (SRH) outcomes and to locate key policy areas where action can address inequalities in health highlighted the importance, of six upstream factors. These were: economic crises and hostile discourse on migration; limited legal entitlements and rights and administrative barriers to their operation; inadequate resources and financial barriers; poor living and working conditions; cultural and linguistic and gender-related barriers; as well as stigma and discrimination. In the present summary of migrant and refugee health in massive migration areas, these upstream factors were seen to be interactive facets that are relevant across many, if not all, areas of physical, mental and social health.

**Human security:** The interactive nature of the seven elements of human security identified in the 1994 UNDP Human Development Report [36] is extensively illustrated by the situations of migrants and refugees in massive migration areas. Their health security is impacted by threats to security of food and their environmental and economic situations, by the physical, economic and environmental security of themselves and their families and by the community and political contexts in which they find themselves. In turn, the health security of the migrants and refugees has implications for these elements of human security for their hosts or countries through which they transit. The significance of this interactivity is starkly highlighted by the COVID-19 pandemic, in which the vulnerability of migrants and refugees, exacerbated not only by their limited entitlements to public health services and health care but also by their lack of other securities such as food, economic and environmental, in turn becomes a vulnerability to health security for all, since no-one is safe until everyone is safe [34].

## 5. Conclusions: Minding the Gaps

The application of an extensive framework, which integrates structural factors, health determinants and a human security perspective, provides a broad approach to the identification of gaps in the health of migrants and refugees in areas of mass migration. Across all these dimensions of the determinants of migrant and refugee health, many areas of gaps are highlighted and signposted for urgent attention.

While migration is a permanent structural factor in human society [13], the large increase in numbers of migrants and refugees in recent years has stimulated pressing international concern. The nature of the responses to this concern has been rooted in historic systems that are intrinsically weak in accountability, highly fragmented and often non-binding, as signified by the phrasing of the UN New York Declaration and its two later-annexed Global Compacts. To date, outcomes have tended to focus on short-term stabilization rather than long-term solutions, but there has been growing recognition of the need to reset the agenda, especially in the light of future migration projections with the climate change and its consequences, including increased risk for new pandemics [136,137].

The advent of COVID-19 has exposed both flaws and dangers in the current approach, particularly with regard to three aspects: (1) the vulnerability of migrants and refugees, which has meant that their health needs have often been very poorly addressed, has generally left them last in the queue for protection from and diagnosis and treatment of COVID-19 infection; (2) the lack of inclusion of migrants and refugees in health services and public health programmes has in turn exposed the general population to increased risks of resurgent waves of the pandemic; and (3) this cross-vulnerability extends far beyond the health sector, impacting on human security elements both for the migrants and refugees and for the general population.

COVID-19 was not the first viral disease to cause major disruptions around the world (others in recent decades have included HIV/AIDS, SARS, MERS, Ebola, Zika and several influenza types) and is not likely to be the last. It has been estimated that 1.7 million currently undiscovered viruses are present in mammal and avian hosts, with up to about half of these potentially having the ability to infect humans [138]. Recognizing and acting on the principle that “no-one is safe until everyone is safe” requires that provisions to include, protect and treat all migrants and refugees must be an essential component of more effective strategies for global health security.

The question going forward is not so much about who is concerned—to which, in a sense, the answer is everyone—but rather that the highly fragmented, inconsistent and poorly accountable mechanisms in place tend not deliver effective policies and programmes. Minding about the gaps needs to translate into a new political and societal willingness to solve deep-seated and long-standing challenges.

The period ahead in the 2020s offers a potentially game-changing opportunity, a cosmopolitan moment [139], to reform the paradigm, due to the conjunction of some major forces. In the immediate aftermath of the COVID-19 pandemic, with the immense human and economic costs of poor preparedness visible to all, there will be determined, worldwide efforts to improve health security globally, rooted in the recognition that health and safety must be seen as a shared global public good. Meanwhile, attention to the SDGs [140] with their bold ambitions, among others, to achieve universal health coverage and leave no-one behind, will be strongly increased in this decade as efforts are intensified to meet the goals and targets of Agenda 2030. Combined with a perspective that underscores the interdependence of all the elements of human security, this confluence of pressures, goals and principles could potentially provide a frame-shift in the treatment of migrants and refugees, including their access to health. If this does not happen, then the broader goals of strong global health security and of fully inclusive sustainable development will also be missed. As another recent meeting of the World Health Summit M8 Alliance Expert Group on Migrant and Refugee Health reported, migrants and refugees, as a group, serve as the ‘canary in the cage’, acting as a litmus test for the competence of the systems in which they are situated [2].

## Data Availability

Not applicable.
